# Role of the IL-6/STAT3 Signaling Axis in the Protective Effect of Selenomethionine Against Zearalenone-Induced Hepatic Inflammatory Injury in Rabbits

**DOI:** 10.3390/toxins17060275

**Published:** 2025-05-30

**Authors:** Xiaoguang Chen, Wenjuan Wei, Haonan Li, Wenjing Xu, Qiongxia Lv, Yumei Liu, Ziqiang Zhang

**Affiliations:** College of Animal Science and Technology, Henan University of Science and Technology, Luoyang 471000, China; wwj3456453421@163.com (W.W.); lihaonan09@163.com (H.L.); xwenjing0223@163.com (W.X.); lvqx20001@163.com (Q.L.); yumeiliu@haust.edu.cn (Y.L.); ziqiangzhang@haust.edu.cn (Z.Z.)

**Keywords:** selenomethionine, zearalenone, hepatic injury, inflammatory cytokines, IL-6/STAT3 signaling axis

## Abstract

Zearalenone (ZEA), a mycotoxin primarily generated by the Fusarium species, constitutes a prevalent contaminant in both human and animal feedstuffs. Chronic exposure to this mycotoxin induces hepatic inflammatory responses in livestock species including rabbits, ultimately leading to organ damage. Selenomethionine (SeMet), an organic selenium source recognized for its antioxidant properties and anti-inflammatory bioactivity, demonstrates protective benefits in animals through its detoxification mechanism and growth promotion. The present study investigated the protective effect of SeMet against ZEA-induced hepatic inflammation and elucidated its underlying mechanisms. Fifty healthy 90-day-old rabbits were randomly divided into five groups: control, ZEA-exposed and three SeMet-supplemented groups receiving 0.2, 0.35 or 0.5 mg/kg via dietary inclusion. After two weeks of SeMet pretreatment, ZEA administration (1.2 mg/kg B.W.) was imitated via oral gavage daily for one week in both the ZEA group and three SeMet-treated groups. As a result, ZEA exposure induced the significant structural disruption of the hepatic lobules, accompanied by increased collagen deposition, elevated pro-inflammatory cytokine profiles (IL-6, IL-1β, TNF-α) and reduced anti-inflammatory mediator levels (IL-10, TGF-β). SeMet supplementation alleviated ZEA-induced histological alterations, including inflammatory cell infiltration and collagen accumulation. Biochemical analysis indicated the restoration of inflammatory markers to near-normal levels when treated with SeMet. Notably, immunohistochemical results showed that SeMet significantly reduced the protein levels of IL-6 and its downstream target STAT3 under ZEA exposure. These findings indicated that SeMet attenuated ZEA-induced hepatic inflammation by modulating the IL-6/STAT3 signaling axis, with dietary supplementation of 0.35 mg/kg SeMet exhibiting the most significant effect on alleviating ZEA-induced hepatic inflammatory injury.

## 1. Introduction

ZEA, a secondary metabolite produced by fungi of the genus *Fusarium*, is a widespread contaminant in cereals including corn, wheat and their by-products [1]. ZEA was initially isolated from moldy corn infected by *Fusarium graminearum* and *Fusarium sporotrichioides*, and its chemical structure was elucidated by Urry in 1966. Moreover, ZEA contamination has expanded beyond traditional cereals to encompass oil and grain products (feed, edible oil, bread) as well as animal-derived foods (meat, eggs, milk). The thermal stability of ZEA is exceptional, maintaining structural integrity during thermal processing. After oral ingestion, this mycotoxin undergoes rapid absorption across the gastrointestinal epithelium, enters systemic circulation and ultimately accumulates in multiple organs, posing health hazards to humans and animals. Apart from its well-characterized endocrine-disrupting activity through estrogen receptor (ER)-mediated signaling, ZEA has been shown to induce oxidative stress, apoptosis and immune suppression in animals [2]. For example, ZEA exposure has been linked to suppressed lymphocyte proliferation and compromised intestinal barrier integrity in piglets [3,4,5,6]. Recent investigations further reveal that this mycotoxin exacerbates hepatic and intestinal inflammation by activating the NF-κB signaling pathway [7,8]. Notably, rabbits, as herbivorous livestock with diets comprising over 60% grains, are particularly susceptible to long-term exposure to ZEA-contaminated environments compared to other species [9]. Studies indicate that ZEA exerts significant toxicity in rabbits by impairing mitochondrial function in hepatocytes and elevating the serum levels of liver enzymes such as Alanine Aminotransferase (ALT) and Aspartate Aminotransferase (AST), indicative of hepatocellular damage [10,11].

Emerging evidence has shed light on the connection between ZEA-induced hepatotoxicity and the dysregulation of inflammatory cascades. AbuZahra et al. demonstrated that ZEA exposure induced the significant upregulation of pro-inflammatory cytokines (IL-6, IL-1β, TNF-α) in murine liver tissue [12]. Further mechanistic studies have indicated that mycotoxins and their metabolites could trigger the hyperphosphorylation of STAT3 in hepatocytes via reactive oxygen species (ROS)-mediated pathways. As a core transcription regulator in IL-6 signaling, sustained STAT3 activation can drive the excessive release of inflammatory mediators, thereby exacerbating liver injury [13,14]. Notably, Liu et al. showed that the pharmacological inhibition of STAT3 phosphorylation using the inhibitor Static significantly attenuated acetaminophen-induced hepatocyte proptosis [15]. These findings also suggest the crucial role of the IL-6/STAT3 axis in hepatotoxicity caused by mold toxins, including ZEA and its metabolites. Current strategies for ZEA toxicity management in rabbit husbandry, including physical adsorbents and microbial degradation [16], primarily focus on limiting toxin absorption, but fail to counteract the ongoing hepatic damage caused by ZEA that has already entered the body. In recent years, significant progress has been made in research on the hepatoprotective and anti-inflammatory regulatory effects of natural compounds. For instance, astaxanthin has been shown to significantly improve the hepatic lipid metabolic disorder caused by microcystin-LR [17]. Chu et al. discovered that graveoline can effectively alleviate D-GalN/LPS-induced acute liver injury by reducing the level of inflammatory factor TNF-α, upregulating the expressions of anti-inflammatory factors IL-4 and IL-10 and inhibiting the JAK1/STAT3 signaling pathway [18]. These studies have provided important references for alleviating ZEA-induced hepatic inflammatory injury.

Current research has highlighted growing interest in organic selenium compounds, such as SeMet and selenium polysaccharides, due to their distinctive biological properties. Among these, as the predominant organic selenium species in biological systems, SeMet, not only shows high bioavailability but also bolsters the body’s capacity to clear ROS through its incorporation into antioxidant enzymes such as glutathione peroxidase (GSH-Px). Currently, organic selenium compounds, such as SeMet and selenium polysaccharides, have attracted considerable attention for their unique biological activities. Among them, as the predominant organic form of selenium in biological systems, SeMet, not only exhibits high bioavailability but also enhances the body’s ability to clear ROS by being incorporated into antioxidant enzymes such as GSH-Px [16]. These characteristics establish SeMet as an important nutritional supplement with significant potential in improving livestock health and production performance, which thereby has a positive impact on the economic benefits of the livestock industry. For instance, Falk et al. found that dietary supplementation with 0.26 and 0.43 mg/kg SeMet could significantly increase body weight gain in weaned piglets [19]. Moreover, appropriate SeMet supplementation was shown to enhance the antioxidant capacity of roosters by upregulating selenoprotein gene expression, thereby protecting the body from damage caused by peroxides and free radicals and ultimately improving the reproductive performance of roosters [20]. The study by Wu et al. also demonstrated that SeMet supplementation can significantly elevate serum selenium content, GSH-Px and SOD activities and total antioxidant capacity in Holstein cows [21]. Given the notable effects of SeMet in enhancing livestock production performance, improving meat quality and augmenting antioxidant capacity, it has been widely promoted and applied in feed supplementation policies. These policies necessitate the establishment of scientific additional standards based on different livestock species and production stages to ensure their safety and efficacy. Emerging studies have expanded our understanding of selenium’s biological roles beyond classical antioxidative functions. Chen et al. identified that organic selenium (including selenoprotein and SeMet) can significantly suppress NF-κB activation, consequently reducing IL-6 levels in heat-stress-challenged murine liver tissues [22]. Complementary findings revealed that selenium nanoparticles could downregulate the activation of the JAK2/STAT3 pathway, thereby decreasing pro-inflammatory cytokine release (IL-1β and IL-6) in murine brain tissue [22]. Additionally, SeMet activates the Nrf2 pathway to strengthen hepatocellular antioxidant capacity, indirectly curbing the overactivation of inflammatory-related pathways [23,24]. These observations provide a theoretical foundation for organic selenium’s hepatoprotective effect against ZEA toxicity, but it remains unclear whether it exerts protective effects by regulating the IL-6/STAT3 signaling pathway in rabbit livers.

To address this issue, the present study employed Hyla rabbits to evaluate the hepatoprotective effect of SeMet against ZEA-induced injury. By establishing a comparative experimental system comprising ZEA exposure and SeMet intervention, supplemented with serum biochemical indicator detection, hepatic histopathological analysis, inflammatory cytokine profiling and inflammatory signaling pathway-specific protein expression assessments, we aimed to elucidate SeMet’s hepatoprotective effects with a particular focus on its interplay with the IL-6/STAT3 signaling axis, thus offering novel insights into the strategies for mitigating ZEA toxicity in rabbit farming.

## 2. Results

### 2.1. Effect of SeMet on Morphological Structure of Liver Tissue in Rabbits Exposed to ZEA

**Figure 1:**
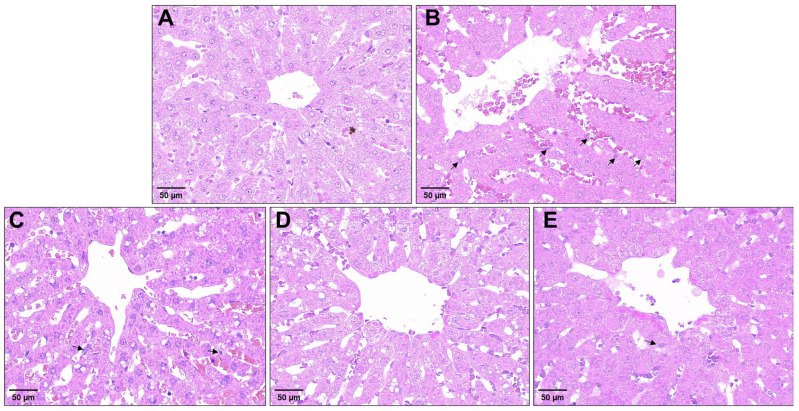
Effects of SeMet on liver tissue structure in rabbits exposed to ZEA. Representative graphs of liver sections stained with HE (20× magnification; scale bar = 50 μm): Black arrows, inflammatory cell infiltration (n = 5). Treatments represented as (**A**) control, (**B**) ZEA-challenged group, (**C**) SeMet-L group, (**D**) SeMet-M group and (**E**) SeMet-H group. Control group exhibited orderly arranged hepatocytes and hepatic sinusoids around central vein and intact structural integrity. ZEA-challenged group displayed disorganized hepatocyte alignment, dilated and congested sinusoids and inflammatory infiltration. Groups pretreated with different doses of SeMet showed notably ameliorated histoarchitectural damage, with most pronounced improvement (*p* < 0.05) in medium-dose group.

To investigate ZEA-induced hepatic damage and the protective role of SeMet, the histological changes in the liver were analyzed using hematoxylin–eosin (HE) staining. As illustrated in Figure 1, the control group exhibited an intact liver architecture with uniform staining, regularly arranged hepatocyte cords, distinct cell membranes and abundant cytoplasm (Figure 1A). In contrast, the ZEA-exposed group displayed a disrupted liver lobular structure characterized by blurred hepatocyte boundaries, dilated and congested sinusoids, inflammatory cell infiltration and even scattered pyknotic nuclei (Figure 1B). After pretreatment with different doses of SeMet, these pathological alterations showed varying degrees of improvement (Figure 1D), with the medium-dose group displaying the most significant amelioration. However, moderate sinusoidal congestion and residual inflammatory infiltration were still observed in low- and high-dose groups (Figure 1C,E).

### 2.2. Effect of SeMet on Inflammatory Injury of Liver Tissue Induced by ZEA

**Figure 2:**
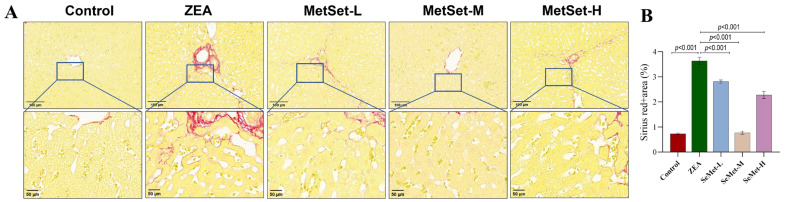
Hepatic fibrosis assessment in rabbit livers. (**A**) Sirius red staining results; (**B**) quantification of collagen fiber content. Sirius red staining revealed elevated collagen deposition in the ZEA-challenged group, predominantly localized to ductule or vascular walls. SeMet pretreatment reduced the accumulation of collagen, with the medium-dose group showing the most significant reduction (*p* < 0.001). Minimal collagen deposition was observed in the control group. Top row: 10× magnification, scale bar = 100 μm. Bottom row: Enlarged views (20×magnification) of boxed regions in the top row, scale bar = 50 μm (n = 5).

The abnormal deposition of collagen fibers, which is regarded as a hallmark of inflammation, was evaluated through Sirius red staining. As depicted in Figure 2A, the hepatic architecture in the control group remained well preserved, with only minimal collagen fiber deposition observed around the vascular and central vein regions. In contrast, the ZEA-challenged group displayed an elevated accumulation of collagen (Figure 2B), indicative of heightened hepatic inflammation, which was further corroborated by a quantitative analysis of the collagen volume fraction (*p* < 0.001). On the other hand, pretreatment with different doses of SeMet led to varying degrees of fibrosis alleviation, reducing it by 22.7%, 78.8% and 37.4%, respectively (Figure 2B, *p* < 0.001). Notably, the medium-dose SeMet group exhibited a markedly reduced collagen distribution, with pathological scoring showing a significantly lower fibrotic area compared to the ZEA group (*p* < 0.001). Although the low- and high-dose groups exhibited a partial reduction in collagen deposition, their alleviating effect, as quantified by fibrosis scoring, was inferior to that of the medium-dose group.

Furthermore, we measured the serum levels of the liver function biomarkers serving as quantitative indicators of hepatic inflammation. As shown in Table 1, compared to the control group, the ZEA-challenged group exhibited marked elevations in albumin (ALB, 54.27 ± 0.15 g/L), globulin (GLOB, 17.63 ± 1.70 g/L) and total bile acid (TBA, 16.04 ± 1.05 μmol/L) (*p* < 0.05), alongside a concurrent decline in the albumin-to-globulin ratio (A/G, 2.95 ± 0.35; *p* < 0.05). Following SeMet pretreatment, these parameters exhibited varying degrees of amelioration. The medium-dose group achieved a near-normalization of ALB level (43.97 ± 1.69 g/L) and a significantly elevated A/G ratio (3.77 ± 0.25; *p* < 0.05) relative to the ZEA group. Although low- and high-dose SeMet partially attenuated ZEA-induced TBA elevation, no statistically significant differences in total bilirubin (TB) level were observed compared to the ZEA-challenged group (*p* > 0.05). Collectively, these data indicated that ZEA could exacerbate hepatic inflammatory injury, whereas medium-dose SeMet showed optimal efficacy in liver function recovery (*p* < 0.05).

### 2.3. Effect of SeMet on Levels of Hepatic Inflammatory Cytokines in ZEA-Exposed Rabbits

**Figure 3:**
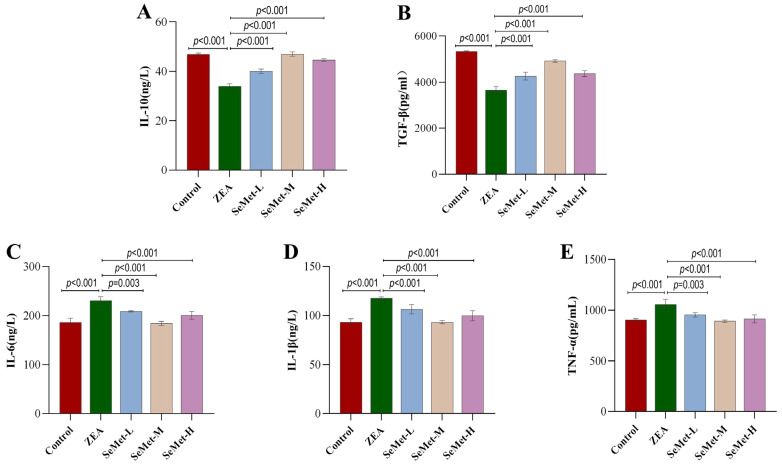
ELISA analysis results of hepatic inflammatory cytokine levels. (**A**–**E**): The contents of interleukin-10 (IL-10), transforming growth factor-β (TGF-β), interleukin-6 (IL-6), interleukin-1β (IL-1β) and tumor necrosis factor-α (TNF-α) in rabbit livers, respectively (n = 5).

It is well-known that inflammatory cytokines exhibit dynamic expression patterns during various types of inflammatory injuries. In this study, hepatic concentrations of anti-inflammatory cytokines (IL-10 and TGF-β) and pro-inflammatory mediators (IL-6, IL-1β and TNF-α) were quantified via ELISA in rabbits across experimental groups. As demonstrated in Figure 3, relative to controls, ZEA exposure significantly suppressed anti-inflammatory cytokine levels, with IL-10 and TGF-β reduced by 27.5% and 31.6%, respectively (*p* < 0.001), Conversely, it markedly upregulated pro-inflammatory cytokines, evidenced by 24.1%, 30.0% and 16.9% elevations in IL-6, IL-1β and TNF-α levels (*p* < 0.001), suggesting that ZEA can induce a hepatic inflammatory response. Notably, the differential restoration of inflammatory cytokine levels was achieved after supplementation with different doses of SeMet, with the medium-dose group exhibiting the more pronounced regulatory effect. Specifically, in the SeMet-M group, the levels of IL-10 and TGF-β were restored by 38.2% and 34.8%, respectively, while the levels of pro-inflammatory cytokines IL-6, IL-1β and TNFα were restored to a baseline range similar to that of the control group (with respective decreases of 20.2%, 20.8% and 15.6%) (*p* < 0.001). Despite improvements in the cytokine profiles (IL-10, IL-6, IL-1β) observed in the high-dose group, high-dose SeMet exhibited an attenuated regulatory potency compared to the medium-dose SeMet. These results indicated that SeMet could alleviate ZEA-triggered hepatic inflammation by the bidirectional modulation of inflammatory cytokines.

### 2.4. Effect of SeMet on the Expressions of IL-6/STAT3 Pathway-Related Genes in ZEA-Exposed Rabbit Livers

**Figure 4:**
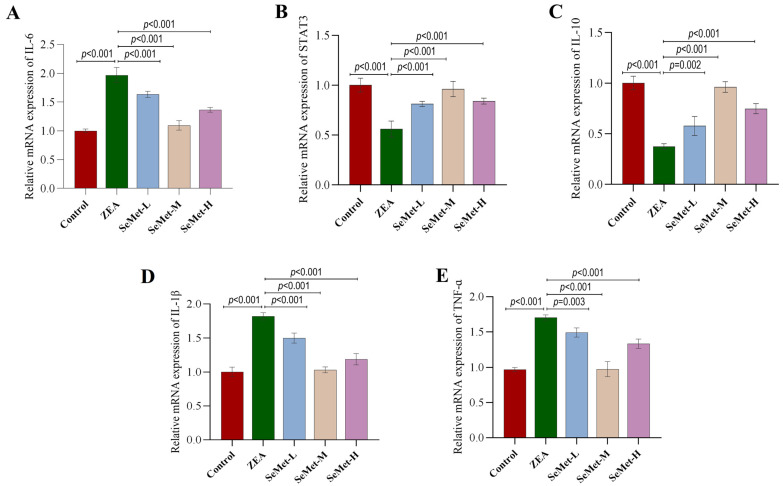
mRNA expression levels of IL-6/STAT3 pathway-related genes in hepatic tissues. (**A**–**E**) The relative mRNA abundances of IL-6, STAT3, IL-10, IL-1β and TNF-α in rabbit livers (n = 5).

To elucidate the mechanism of the SeMet-mediated mitigation of hepatic inflammatory injury induced by ZEA, qPCR analysis was employed to quantify transcriptional profiles of IL-6/STAT3 pathway-related genes in rabbit livers. As shown in Figure 4, compared with the control group, the mRNA level of IL-6 (an upstream component of the pathway) in the livers of rabbits exposed to ZEA significantly increased by 96.7% (*p* < 0.001), while the mRNA level of its downstream effector STAT3 significantly decreased by 76.8% (*p* < 0.05). Meanwhile, the mRNA level of the anti-inflammatory factor IL-10 regulated by STAT3 also significantly decreased by 62.8%, whereas the mRNA levels of the pro-inflammatory factors IL-1β and TNFα showed the opposite trend, significantly increasing by 81.7% and 12.5%, respectively (*p* < 0.001). After pretreatment with SeMet, the transcriptional levels of these factors were reversed to varying degrees, with the medium dose of SeMet showing a more significant regulatory effect. Specifically, compared to the ZEA group, the mRNA level of IL-6 significantly decreased by 16.9%, 44.4% and 30.6%, respectively (*p* < 0.001), with the medium-dose group showing the most significant reduction. The mRNA levels of STAT3 and its downstream target IL-10 significantly recovered, respectively (*p* < 0.05), while the transcriptional levels of IL-1β and TNF-α were reduced to levels comparable to those of the control group (*p* < 0.05). In comparison, the effects of low-dose and high-dose SeMet were less pronounced than those of the medium dose.

### 2.5. Effects of SeMet on Expression and Location of Hepatic IL-6/STAT3 Pathway-Involved Proteins in ZEA-Exposed Rabbits

**Figure 5:**
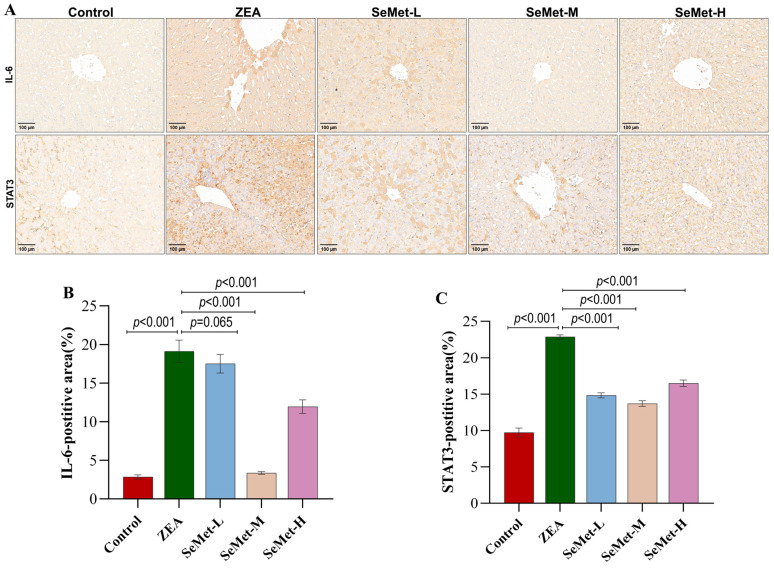
Immunohistochemistry analysis of IL-6 and STAT3 proteins in rabbit livers after ZEA exposure. (**A**) Representative photomicrograph showing IL-6 and STAT3 immunohistochemical staining. (**B**,**C**) Quantification of IL-6 and STAT3 protein-positive areas, respectively. Magnification: 10×; scale bar: 100 μm (n = 5).

This present study further examined the expression and localization of key proteins in the IL-6/STAT3 pathway using immunohistochemical (IHC) methods. As illustrated in Figure 5, the control specimens exhibited negligible immunoreactivity for IL-6 and STAT3, appearing as faint yellow-brown signals. In contrast, ZEA-exposed livers displayed intense granular immunostaining for both proteins, with deep-yellow signals concentrated in the cells around the portal area and central vein region. Quantitative IHC assessment confirmed the significantly elevated expression of both proteins in the ZEA group compared to the control group (*p* < 0.001). After pretreatment with SeMet, the immunostaining intensities for both proteins showed varying degrees of reduction. Specifically, medium-dose SeMet displayed a more pronounced effect, with a marked reduction in staining (*p* < 0.001), which approached but did not fully restore the control levels, which demonstrated that a certain concentration of SeMet could counteract the dysregulation of IL-6/STAT3 pathway-related proteins caused by ZEA.

## 3. Discussion

ZEA, a mycotoxin ubiquitously present in mold-contaminated grains, has drawn scientific interest due to its toxic effects, particularly its damaging impact on the liver [25]. As the central metabolic organ responsible for detoxification and immune regulation, liver dysfunction invariably accompanies systemic inflammatory responses and metabolic disturbances [26]. Recent studies have highlighted the potential of SeMet in alleviating environmental-toxin-induced liver damage, owing to its distinctive antioxidant and anti-inflammatory capabilities. Nevertheless, ZEA’s impact on rabbit hepatic immune function, along with SeMet’s underlying intervention mechanism, remains insufficiently characterized. To address this, our study conducted a systematic analysis of ZEA-induced hepatic pathology in rabbits and SeMet’s protective potential. Notably, preliminary data indicated that SeMet effectively attenuated ZEA-induced hepatic inflammation, potentially through suppressing the IL-6/STAT3 signaling cascade.

The hepatotoxicity of ZEA primarily manifests through its interference with the immune system. Our findings demonstrated that ZEA exposure significantly elevated serum GLOB and ALB levels concomitantly with the reduced A/G ratio, which aligned with Alassane-Kpembi et al.’s report that mycotoxins stimulate immune cells to release acute-phase proteins (e.g., GLOB) while inhibiting the synthetic function of the liver [27]. Notably, the observed elevation in ALB level likely represented hemoconcentration secondary to inflammation-induced vascular hyperpermeability, rather than the enhanced hepatic function. Moreover, the significant increase in TBA levels suggested the ZEA-induced disruption of bile acid metabolism, potentially attributable to either the impairment of hepatocyte membrane integrity or the downregulation of bile acid transporters [28]. These alterations in biomarkers collectively underscored ZEA’s ability to destabilize hepatic metabolic and immune homeostasis, providing critical insights for the further investigation of its inflammatory effects.

It is well-established that alterations in the hepatic architecture provide direct morphological evidence of inflammatory injury severity. HE staining confirmed that ZEA exposure induced the disorganization of lobular structure, accompanied by inflammatory cell infiltration and nuclear pyknosis, aligning with typical features of hepatitis-like pathology [29,30]. Consistently, Sirius red staining revealed the aberrant deposition of collagen fibers, indicating ZEA’s fibrogenesis-promoting activity. As fibrosis represents the ultimate pathological consequence of chronic inflammation, its development is mechanistically associated with the sustained release of pro-inflammatory mediators like TNF-α and IL-6 [31]. In this study, a significantly elevated collagen volume fraction was observed in the ZEA group, indicating the initiation of liver fibrosis. Importantly, SeMet pretreatment markedly attenuated collagen deposition, with the medium-dose group exhibiting optimal effects. This protective effect parallels Zhang et al.’s findings on the suppression of hepatic stellate cell activation by selenium compounds [32], suggesting that SeMet may exert protective effects through disrupting the inflammation–fibrosis cascade.

The dysregulation of inflammatory cytokines constitutes a pivotal mechanism underlying ZEA-induced hepatic injury [33]. ELISA data revealed that ZEA markedly suppressed anti-inflammatory cytokines (IL-10, TGF-β) while upregulating pro-inflammatory mediators (IL-6, IL-1β, TNF-α), suggesting that ZEA may induce liver damage through the activation of the canonical inflammatory pathway. This observation corroborates Xia et al.’s report that ZEA promoted the release of pro-inflammatory cytokines via activating the NF-κB pathway, with concurrent anti-inflammatory suppression exacerbating inflammatory progression [8]. Intriguingly, SeMet supplementation exhibited a bidirectional immunomodulatory effect: the medium-dose SeMet not only restored anti-inflammatory cytokine levels but also significantly attenuated pro-inflammatory overexpression. This aligns with previous evidence that selenium-containing proteins (e.g., GSH-Px, TrxR) modulate NF-κB signaling [34]. These results highlighted the critical influence of SeMet dosing on the protective effect.

Considering these observations, this study centered on the IL-6/STAT3 signaling axis, a central hub in inflammatory regulation, to further dissect the mechanistic basis of SeMet’s protective effect. qPCR analysis detected a significant upregulation of IL-6 mRNA while there was a paradoxical reduction in STAT3 transcripts following ZEA exposure. This contradictory phenomenon likely arises from negative feedback regulation, wherein IL-6 hyperactivation inhibits STAT3 transcription via the induction of SOCS3 expression, or alternatively, reflects the post-transcriptional regulatory mechanism of STAT3 [35]. One study in colorectal cancer (CRC) found that the STAT3 protein level was significantly increased despite the unchanged mRNA expression, suggesting the disruption of the normal translational regulation of the STAT3 protein during pathological progression [36]. Further supporting this mechanistic dissociation, the study by Edsbäcker et al. also demonstrated that phosphorylation status and protein levels of STAT3 were predominantly modulated through various upstream signaling cascades [37]. Studies have revealed that the binding of IL-6 to its membrane receptor (IL-6R) activated the JAK/STAT3 pathway through phosphorylation rather than the transcriptional upregulation of STAT3 [38]. Our immunohistochemical analysis also provided corroborating evidence: the ectopic nuclear accumulation of STAT3 protein in ZEA-treated livers signified the hyperactivation of this pathway, thereby stimulating IL-6 overexpression through a self-reinforcing feedback loop [39]. This pathological circuit may account for the concurrent decline in STAT3 mRNA levels and the significant increase in downstream pro-inflammatory cytokines (IL-1β, TNF-α). Remarkably, SeMet pretreatment counteracted the pathological hyperactivation of the IL-6/STAT3 pathway. Of particular significance, medium-dose SeMet exerted an optimal anti-inflammatory effect by suppressing IL-6 overexpression, subsequently restoring STAT3 homeostasis and downregulating pro-inflammatory transcripts. These findings aligned with Swetha et al.’s report on the selenium-mediated regulation of STAT3 phosphorylation in a severe acute pancreatitis mouse model [40], suggesting that SeMet’s protection might involve post-translational modifications of STAT3 rather than direct transcriptional regulation.

It is noteworthy that the protective effect of SeMet exhibited a non-linear dose–response relationship. The low-dose SeMet partially alleviated cytokine dysregulation, demonstrating its limited antifibrotic effect. Conversely, the high-dose group also showed attenuated immune modulation, potentially attributable to selenium’s “double-edged sword” effect, where excessive selenium intake can interfere with selenoprotein synthesis, paradoxically inducing oxidative stress, and partially counteracting its anti-inflammatory efficacy [32]. However, these are only our speculations, and the exact reasons still need further investigation. Additionally, the current study primarily focused on the mechanisms of liver injury caused by ZEA during the acute exposure phase and the intervention effects of SeMet. The exposure time to ZEA was relatively short, and there was a lack of follow-up on long-term outcomes. In future studies, we will consider extending the exposure time to ZEA and tracking long-term outcomes to more comprehensively evaluate the chronic toxic effects of ZEA and the long-term intervention effects of SeMet. Moreover, this study did not delve into the regulatory crosstalk between SeMet and parallel inflammatory pathways (such as NF-κB, MAPK, etc.). This requires a systematic analysis of their network-level mechanisms in the future.

## 4. Conclusions

This study elucidated the molecular mechanism underlying ZEA-induced hepatic inflammatory injury in rabbits and verified the hepatoprotective effect of SeMet by modulating the IL-6/STAT3 signaling axis. Notably, the most pronounced anti-inflammatory effect was achieved with the dietary supplementation of 0.35 mg/kg SeMet. These findings underscore the importance of SeMet dosage optimization in animated animal husbandry to minimize adverse effects while maximizing protective benefits, thereby providing both a theoretical basis for alleviating ZEA toxicity and practical guidelines for selenium-based preparations in hepatoprotection. Moreover, the SeMet dosage determined in this study serves as a validated reference for production applications, which is conducive to enhancing the economic benefits and improving welfare standards in livestock husbandry.

## 5. Materials and Methods

### 5.1. Chemical Reagents

ZEA was procured from Alta Scientific Co., Ltd. (Tianjin, China), while SeMet was sourced from Sangon Biotech (Shanghai, China). High-purity olive oil was obtained from Olivela (Italy). Fixation reagents including paraformaldehyde (4%), glutaraldehyde (2.5%) and sodium pentobarbital (3%) were purchased from Wuhan Duolaibi Biotechnology Co., Ltd. (Wuhan, China). Molecular biology reagents comprising the BCA Protein Quantification Kit (p0012s), cDNA Synthesis Kit (G3330-100) and SYBR^®^ Premix Ex Taq™ Kit (G3326-15) were acquired from Wuhan Saibainuo Biotechnology Co., Ltd. (Wuhan, China). Histological and immunological materials, specifically the Sirius Red Staining Kit (G1078-100ML), polyclonal rabbit anti-IL-6 (GB11117), anti-STAT3 (GB150001) and HRP-conjugated goat anti-rabbit secondary antibody (GB23303) were supplied by Servicebio (Wuhan, China). ELISA kits for IL-10 (ml027828), TGF-β (ml036722), IL-6 (ml027844), IL-1β (ml027836) and TNF-α (ml028087) were from Shanghai Enzyme-Linked Biotechnology Co., Ltd. (Shanghai, China).

### 5.2. Animal Grouping and Treatment

**Figure 6:**
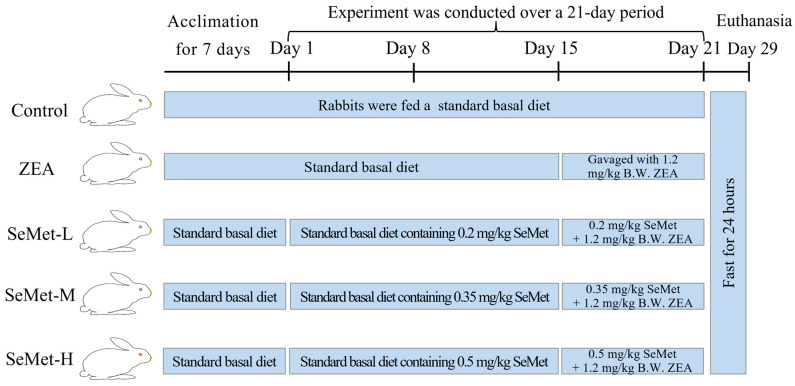
The scheme outlining the experimental design for animal grouping and drug administration (n = 5).

Healthy ninety-day-old Hyla rabbits (3.5 ± 0.1 kg) obtained from the Experimental Animal Center of Henan University of Science and Technology (Luoyang, China) underwent seven-day acclimatization under controlled conditions (21–25 °C, 50–70% relative humidity). Following acclimation, animals were randomly allocated into five groups (ten per group, with an equal number of male and female rabbits): control, ZEA, low-dose SeMet (SeMet-L), medium-dose SeMet (SeMet-M) and high-dose SeMet (SeMet-H). Throughout the trial, each rabbit was provided with 200 g of a basal diet daily (compositions are detailed in Table S1) along with ad libitum water access. Throughout the three-week experimental phase, SeMet was administrated at concentrations of 0.2, 0.35 and 0.5 mg/kg basal diets to the respective treatment groups [33]. Beginning in the third experimental week, the ZEA and three SeMet groups were administered with 0.5 mL olive oil containing 1.2 mg/kg B.W. ZEA via oral gavage daily for one week [34], whereas the control group was administered equivalent volumes of olive oil (Figure 6). All animal care and experimental procedures strictly adhered to institutional guidelines and national regulations, with formal approval from the Institutional Animal Care and Use Committee (IACUC) of Henan University of Science and Technology (China) (Approval No. 20190619024).

### 5.3. Collection of Liver Tissue Samples

Following 24 h of the final dose administration, rabbits were anesthetized by the intravenous injection of 3% sodium pentobarbital through the marginal ear vein. Blood samples were collected via cardiac puncture into anticoagulant tubes, followed by centrifugation at 3000× *g* for 15 min (Low Journal Pre-Protect 7 Centrifuge, Hunan Xiangyi Instrument Co., Xiangtan, China). Blood samples were collected via cardiac puncture into anticoagulant tubes and centrifuged at 3000× *g* for 15 min (Low Journal Pre-Protect 7 Centrifuge, Hunan Xiangyi Instrument Co., China). The resulting plasma supernatants were collected and stored at −20 °C for subsequent analysis. After euthanasia, liver tissues were harvested, with one portion fixed in 4% paraformaldehyde for subsequent paraffin embedding, and the remaining tissue was snap-frozen at −80 °C for future assays.

### 5.4. Serum Biochemical Analysis

Serum samples previously stored at −20 °C were taken and thawed on an ice pack. Subsequently, the concentrations of serum albumin (ALB), globulin (GLOB), total bilirubin (TB), total bile acid (TBA) along with the albumin-to-globulin ratio (A/G) in the serum of each group were quantified using a Mindray BS-240Vet automated biochemical analyzer (Shenzhen, China) for all experimental groups [34].

### 5.5. HE and Sirius Red Staining for Hepatic Histopathology Analysis

Liver tissue specimens were fixed in 4% paraformaldehyde for 24 h, followed by undergoing continuous rinsing under running water for 12 h. Tissue processing included sequential dehydration through a graded ethanol series (70% for 40 min, 80% for 1 h, 90% for 1 h, 95% for 40 min and 100% for 1 h), immersion in xylene for transparency (twice, 20 min each) and infiltration with paraffin at 58 °C for 3 h. Using a rotary microtome (Leica RM2016, Chengdu), tissues were sectioned at a 5 μm thickness, floated on a 40 °C water bath to remove wrinkles, mounted onto glass slides and air-dried overnight at 37 °C. For histological evaluation, sections were deparaffinized in xylene (2 × 5 min) and rehydrated through graded ethanol (70–100%) before staining. HE staining was performed by 10 min hematoxylin immersion, differentiation in 1% acid alcohol, blue in 0.2% ammonia water and 1 min eosin counterstaining. Sirius red staining (0.1% Sirius red in saturated picric acid solution, 1 h) was conducted following the manufacturer’s protocol (Servicebio, G1078). After dehydration and clearing, the stained sections were cover-slipped with neutral balsam and visualized under a Nikon ECLIPSE C1 light microscope equipped with a DS-Fi3 camera. Sirius red-positive areas, indicative of collagen deposition, were quantified using Image J software (ImageJ 1.53k) with threshold-based segmentation [35].

### 5.6. ELISA Detection of Inflammation Markers

A total of 0.5 g of frozen liver tissue was mechanically homogenized in PBS (pH 7.4, 1:9 weight/volume ratio) with a tissue grinder, followed by centrifugation at 3000 rpm for 20 min (4 °C). Subsequently, the top 10% of the supernatant was carefully aliquoted. The concentrations of interleukin-6 (IL-6), tumor necrosis factor-α (TNF-α), transforming growth factor-β (TGF-β), interleukin-1β (IL-1β) and interleukin-10 (IL-10) were determined using commercial ELISA kits, strictly adhering to the manufacturer’s instructions. Each assay was conducted with five replicate samples to ensure accuracy.

### 5.7. Quantitative Real-Time PCR Analysis

According to a previously established laboratory protocol, total RNA was extracted from frozen tissues, reverse-transcribed into cDNA using a commercial cDNA Synthesis Kit and subsequently amplified with the SYBR^®^ Premix Ex Taq™ kit. The relative mRNA expression levels of IL-6, STAT3, IL-10, IL-1β and TNF-α in liver samples were quantified using the 2^−ΔΔ^Ct method, normalized to β-actin as the internal reference gene. The corresponding primer sequences are detailed in Table 2.

### 5.8. Immunohistochemical Detection of IL-6 and STAT3 Protein Expression

The harvested liver tissue samples were fixed in 4% formaldehyde for 24 h, followed by being washed under running tap water for 12 h to remove residual fixative. Paraffin sections were prepared following the protocol detailed in the Materials and Methods Section 5.5. For antigen retrieval, the paraffin sections were immersed in citrate buffer (pH 6.0), heated to 100 °C for 15 min using a microwave oven then cooled gradually to room temperature. To block endogenous peroxidase activity, sections were immersed in 3% hydrogen peroxide solution for 25 min at room temperature in the dark. Immunostaining was performed using rabbit polyclonal primary antibodies against IL-6 and STAT3 (1:500 dilutions in 5% skim milk), which were applied to sections and incubated overnight at 4 °C in a humidified chamber. After thorough washing, sections were incubated with HRP-conjugated goat anti-rabbit secondary antibody (1:200 dilution) at 37 °C for 50 min (antibody specifications were provided in Table S2). Colorimetric detection was achieved using 3,3′-diaminobenzidine (DAB) as the chromogen, with the reaction monitored microscopically and halted by rinsing with tap water. For nuclear counterstaining, sections were briefly immersed in hematoxylin, differentiated in acid alcohol and blued in tap water. Final processing included dehydration through graded ethanol, clearing in xylene and mounting with resin-based mounting medium. Digital images were captured under a bright-field microscope. Quantitative analysis of IL-6 and STAT3 immunopositivity was performed by measuring the mean optical density of positive staining using Image J software.

### 5.9. Statistical Analysis

Statistical analysis of the experimental data was performed using one-way analysis of variance (ANOVA) within SPSS 20.0 software. Differences between groups were assessed with the LSD post hoc test for multiple comparisons. All quantitative results were presented as mean ± standard deviation (SD). A *p*-value threshold of *p* < 0.05 was indicative of statistically significant differences between groups [41].

## Figures and Tables

**Table 1:** Hepatic function indices across experimental groups.

Grouping	Liver Function Index				
	ALB (g/L)	GLOB (g/L)	TBA (μmol/L)	TB (μmol/L)	A/G
Control	43.80 ± 3.44 ^b^	13.40 ± 0.10 ^c^	11.57 ± 0.14 ^b^	0.10 ± 0.00	3.74 ± 0.27 ^a^
ZEA	54.27 ± 0.15 ^a^	17.63 ± 1.70 ^a^	16.04 ± 1.05 ^a^	0.73 ± 1.10	2.95 ± 0.35 ^b^
SeMet-L	52.10 ± 1.44 ^a^	16.07 ± 0.76 ^a,b^	13.82 ± 1.14 ^a,b^	0.10 ± 0.00	3.16 ± 0.46 ^a,b^
SeMet-M	43.97 ± 1.69 ^b^	14.53 ± 0.47 ^b,c^	12.03 ± 0.47 ^b^	0.57 ± 0.81	3.77 ± 0.25 ^a^
SeMet-H	51.23 ± 1.43 ^a^	15.20 ± 0.75 ^b,c^	13.53 ± 2.17 ^b^	0.13 ± 0.06	3.37 ± 0.26 ^a,b^

Note: When the letters (a–c) are the same, it indicates that there is no significant difference between the groups (*p* > 0.05). When the letters are different, it indicates that there is a significant difference between the groups (*p* < 0.05). Specifically, when two groups of data are labeled with different letters (e.g., one is labeled as “a” and the other as “b”), this signifies that there is a significant difference between these two groups (*p* < 0.05). Conversely, when two groups of data are labeled with the same letter (e.g., both are labeled as “a”), this indicates that there is no significant difference between these two groups (*p* > 0.05).

**Table 2:** Primers used in the gene expression analysis.

Gene	Sequence (5′ → 3′)
β-Actin	F: CGTGCGGGACATCAAGGAG R: AGGAAGGAGGGCTGGAAGAG
IL-6	F: ACCTGCCTGCTGAGAATCAC R: TCGTCACTCCTGAACTTGGC
STAT3	F: CAGCCTGTCTGCAGAGTTCA R: AAGGTGATCAGGTGCAGCTC
IL-1β	F: AAGACGATAAACCTACCCTGC R: GACTCAAATTCCAGCTTGTCC
TNF-α	F: AAGAGTCCCCAAACAACCTCC R: CTCCACTTGCGGGTTTGCTAC

## Data Availability

The original contributions presented in this study are included in the article/Supplementary Material. Further inquiries can be directed at the corresponding author(s).

## Supplementary Materials

The following supporting information can be downloaded at: https://www.mdpi.com/article/10.3390/toxins17060275/s1, Table S1: Ingredients and chemical composition of basal diets; Table S2: Primary antibody information.
